# Is Elective Implant Removal after Fracture Healing Beneficial? – A Prospective Cohort Study

**DOI:** 10.5704/MOJ.2211.010

**Published:** 2022-11

**Authors:** W Yuan, THI Chua, EBK Kwek

**Affiliations:** 1 Department of Orthopaedic Surgery, Woodlands Health Campus, Singapore; 2 Department of Orthopaedic Surgery, Tan Tock Seng Hospital, Singapore

**Keywords:** implant removal, cold weather pain, fracture

## Abstract

**Introduction:**

Elective implant removal following healed extremity fractures remains controversial. This study aimed to evaluate the reasons and outcomes of implant removal after uneventful healing of limb fractures.

**Materials and methods:**

This is a prospective single-centre observational cohort study. Patients who sustained upper or lower extremity fractures that were fixed and healed uneventfully were included in the study when they elected to remove the implants. Patients were followed for six months post-operatively. Outcomes were assessed with patient satisfaction, symptoms resolution, and complications.

**Results:**

A total of 43 patients were recruited from October 2016 to March 2019. Thirty-six patients (37 implants) were symptomatic. Pain and prominence were the most common complaints, present in 59.5% and 33.3% of patients, respectively. Cold weather pain was also not uncommon (19.0%). Pain improved in 91.3% of the patients who complained of pain. The 94.6% symptomatic patients had at least partial resolution of pre-operative symptoms. All the patients who completed follow-up were satisfied with the procedure. In two patients, there were broken and retained screws intra-operatively. Post-operative complication rate was 23.8%, although no major complications occurred.

**Conclusions:**

Implant removal after uneventful healing of extremity fractures is a safe procedure that conferred a predictable relief of symptoms and satisfactory outcomes in most.

## Introduction

Implant removal is a frequently performed orthopaedic surgery and has been reported to contribute to almost 30% of all elective orthopaedic operations and 15% of all operations^1^. In patients for whom their fractures were fixed with implants, it is controversial whether routine implant removal is required after uneventful fracture healing. There are currently no guidelines or evidence-based recommendations for this practice. Patients may request for implant removal for a variety of reasons: prominence, pain including cold weather pain, or stiffness. However, it is difficult to link the causality of these symptoms to the implants and is unclear if routine implant removal will resolve the symptoms. Moreover, going through another surgery is not without risks, such as retained implants, iatrogenic fractures, or neurovascular injuries.

In studies examining the outcomes of elective implant removal, some showed improved functional outcomes, quality of life and satisfaction after implant removal^2,3^. However, there were concerns about the high early postoperative complication rate^4^. A recent systematic review failed to conclude the effectiveness of elective removal of implants^5^.

Most studies on the outcomes of implant removal are retrospective with small study populations^6-9^. We therefore designed a prospective study aiming to evaluate the reasons and outcomes of implant removal in healed extremity fractures after implant fixation.

## Materials and Methods

This is a prospective single-centre observational cohort study performed in a tertiary trauma centre over three years. Ethical approval was obtained from the institutional review board prior to initiating the study (National Healthcare Group Domain Specific Review Board Reference No. 2015/00769, dated 10 Sep 2015). Patients undergoing elective implant removal were invited to participate in the study if the following inclusion criteria were fulfilled: (1) the patients chose to have their implants removed which was not scheduled as part of the management plan; (2) aged 19 to 75 years.

The exclusion criteria included: (1) patients with osteomyelitis or infected implants; (2) patients scheduled for revision surgery due to implant failure or peri-implant fractures; (3) patients scheduled for implant removal before weight-bearing (e.g. syndesmotic screw); (4) spine implants, finger and metacarpal implants, osteotomy implants, and external fixators; (5) patients who had secondary procedures done as part of the implant removal process, e.g., tenolysis, nerve release, or extensive adhesiolysis. Patients who had multiple implants removed from different locations in the same sitting were not excluded.

The patients were identified from the specialist clinics during their routine follow-ups and eligible patients invited to participate in the study. Informed written consent was obtained for all enrolled patients.

The records of the initial injury and surgery were reviewed for the mechanism of injury, and the presence of open fractures. The interval between initial surgery to implant removal, the status of fracture healing, reason(s) for implant removal, and pain score were documented pre-operatively. Surgeon's training level, type of implants removed, and the presence of intra-operative complications were recorded on the day of the surgery.

Patients were scheduled for a follow-up visit at two weeks, and either visits or telephonic assessment at three and six months post-operatively to assess outcomes and adverse events. Radiographs were taken during the first postoperative visit to look for retained implants, fractures, and healing complications. Patients' satisfaction was assessed with two questions: Are you satisfied with the implant removal? And would you still choose to remove the implants if you were put in the same situation? Pain was assessed using the visual analog scale (VAS). Other outcome measures were resolution of pre-operative symptoms and complications, which including hypertrophic scar/keloid, infection, neurovascular injury, hematoma formation, retained implant(s), iatrogenic fracture, and re-fractures.

## Results

**Fig. 1.**
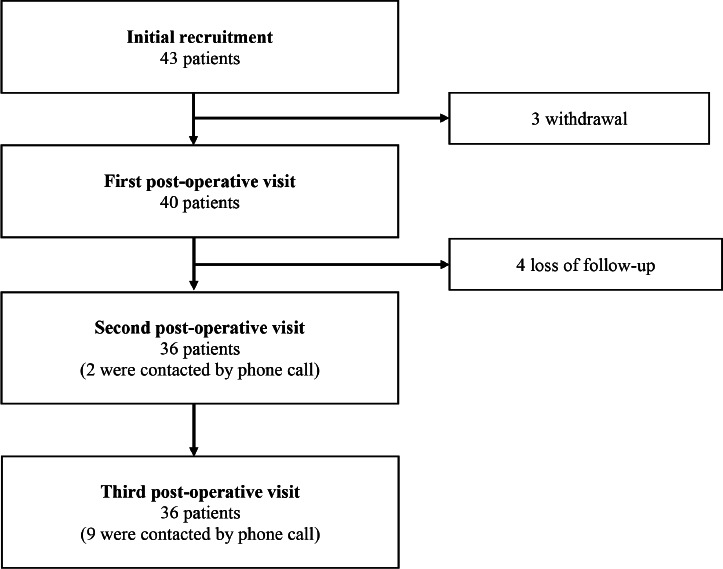
Flowchart of patient recruitment.

A total of 43 patients with 45 implant removal surgeries were recruited for the study (Fig. 1). Two patients underwent two implant removal surgeries of different sites in the same setting. Three patients withdrew voluntarily from the study post-surgery. Of the remaining 40 patients (42 implant removal surgeries), 4 were lost to follow-up after the first post-operative visit. The available data of these four patients were still used for analysis. Thirty-six patients completed the study.

Fifteen patients were female, and 25 were male. The mean age was 40.2 years (range: 19–74 years) at the time of implant removal. Twenty-two patients participated in recreational sports pre-operatively, and the rest were not active in sports. Twenty-eight patients were healthy without any co-morbidities. The rest had well-controlled medical conditions. Seven patients were smokers.

The initial fractures were all closed. They were at the ankle in 16 cases, clavicle in 9, olecranon in 5, tibia/fibula in 3, distal radius in 3, proximal humerus in 3, distal humerus in one, ulnar in one, and distal femur in one. 17 fractures resulted from road traffic accidents, 14 from sports, 8 were falls from standing height, and 3 were falls from height. Preoperative radiographs showed union of all the fractures.

Thirty-six patients (37 implants) were symptomatic. Fifteen patients (37.5%) had multiple complaints. Pain was the most common reason for implant removal. It was present in 25 (59.5%) cases with a mean pre-operative VAS of 3.6 (range: 1–7). Eight (19.0%) cases had pain or discomfort in cold weather or environment. Fourteen (33.3%) implants were removed due to prominence. Other reasons were less common. Four (9.5%) patients complained of stiffness. Three (7.1%) patients felt discomfort at the surgical site without pain. Two (4.8%) patients complained of paraesthesia at the surgical site. One (2.4%) opted for implant removal to avoid triggering airport scanners when travelling. Five (12.0%) expressed a preference to have the implants removed despite being asymptomatic.

The mean time from initial surgery to implant removal was 18.0 months (range: 8–45 months). The implants removed were: 21 locking plates, 18 non-locking plates, 2 intramedullary nails, and cancellous screws for an isolated medial malleolus fracture. Tightropes for syndesmosis fixation in three cases and medial malleolus cancellous screws in two cases were also removed together with the ankle plates.

Of the 25 cases whose implants were removed for pain, 23 completed follow-ups. At 6 months post-operatively, pain improved in 21 (91.3%) cases, including 19 (82.6%) with complete resolution. Average VAS score improved from 3.7 to 0.8. Another patient’s pain score remained the same, but the pain was less constant. She had a medial malleolar fracture which was fixed with two cancellous screws. The pain was worse in one patient whose proximal humerus locking plate was removed, which was due to avascular necrosis of the humeral head. The average VAS score of the 23 patients at final follow-up was 0.8 (range: 0-7). Interestingly, cold weather pain or discomfort resolved in all eight patients. One asymptomatic patient with a clavicle fracture fixed with a reconstruction plate developed pain after implant removal, and his VAS was four at the final follow-up.

A total of 94.6% (35/37) symptomatic cases experienced at least partial resolution of the symptoms. Two patients' symptoms did not improve. They are the above-mentioned patients who had persisting kneeling difficulty kneeling and humeral head avascular necrosis.

At the final follow-up, all 38 patients did not express any regret regarding the procedure. However, one patient (2.6%) was not satisfied with the results. He had a proximal tibia fracture which was fixed with a proximal lateral tibial locking plate and had difficulty kneeling after fixation which did not improve with implant removal despite complete resolution of pain. However, he was not disappointed with his decision for the procedure as he did not wish to have any retained implants.

**Fig. 2.**
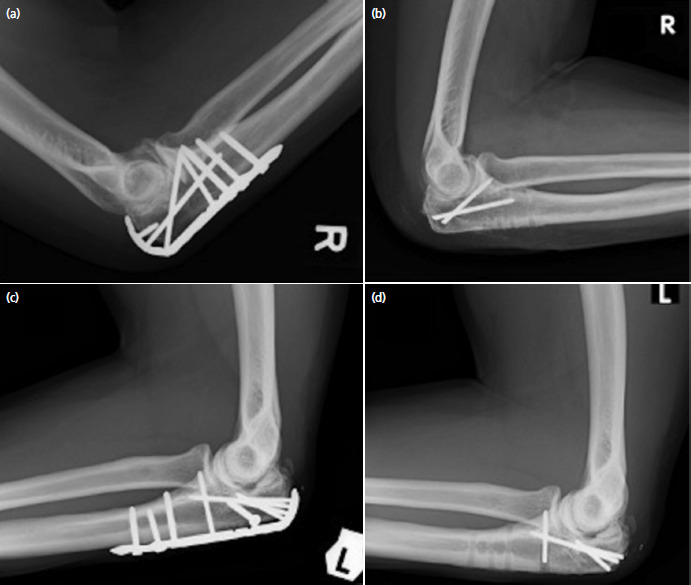
Two cases with broken screws. (a) Pre-operative radiograph showing a healed olecranon fracture with a locking plate in situ. (b) Post-operative radiograph showing two broken and retained locking screws. (c) Pre-operative radiograph of another patient showing a healed olecranon fracture with a locking plate in situ. (d) Post-operative radiograph showing four broken locking screws.

Intra-operative complications occurred in two cases. Both were olecranon fracture fixed with an anatomical locking plate. The interval between initial fixation and implant removal were 17 and 26 months. Two and four 2.7mm locking screws were broken during implant removal and were left retained (Fig. 2). No major intra-operative difficulty was encountered in the rest of the cases.

Post-operative radiographs showed complete removal of implants except for the two cases with broken screws mentioned above. No perioperative fractures occurred.

The overall post-operative complication rate was 23.8% (10/42). These were all minor, including hypertrophic scar in 5, neuropraxia in 3, hematoma formation requiring aspiration in 1, and stitch abscess in 1. None required surgical intervention. Of the three patients with neuropraxia, one had a clavicle fracture and the other two had a distal radius fracture. They had infraclavicular nerve and palmar cutaneous branch of the medial nerve neuropraxia. All were improving at the final follow-up, with one recovering fully. The stitches abscess was treated with oral antibiotics. However, an asymptomatic keloid formed at the infection site. Intra-operative or post-operative refracture did not occur in any of the patients.

## Discussion

Our study found that the most common reasons for implant removal were pain and prominence, followed by cold weather pain or discomfort. Outcomes of implant removal were satisfactory in most patients. Symptoms improved in 94.6% of patients and 97.4% were satisfied with results. Although the complication rate appeared to be high, there were no major complications, and none required surgical intervention. Implant removal is a safe procedure in our series.

Implant removal has been advocated in the past due to concerns about corrosion and carcinogenesis^10^. However, with the advancement of metallurgy and understanding of the correlation between implants and cancer, these risks are now considered as minimal or even non-existent. Currently, indications for removal are mainly "relative" and patient-driven^10^. In our institution, we do not advocate routine implant removal. This explains why most patients who requested for implant removal in our study were symptomatic.

The outcomes of implant removal in the literature are inconsistent. Most studies are from western populations. In a study of 22 patients who underwent implant removal for pain and impingement on the skin following patellar fixation, 86.4% (19/22) had partial or complete resolution of symptoms^11^. Sidky *et al*^8^ found 72.2% (13/18) of the patients’ symptoms improved after removal of tibial nails in his retrospective study. And 77.8% (14/18) would have their implants removed if they were in the same situation. Another prospective study of 59 patients who underwent implant removal of proximal humerus locking plate showed significant improvement of Constant score and pain at three and six months post-operatively. Of the 42 patients who answered the questionnaire at 6 months post-operatively, all were satisfied with implant removal and would choose implant removal again if they were in the same situation^7^. However, in retrospective series of 72 tibial nail removal in 71 patients reported by Karladani *et al*^9^, the pain was reduced in only 39 (54.9%). and only 61.8% (42/68) of patients were satisfied with results. Brown *et al*^12^ reported pain relief in only 50% (11/22) of symptomatic patients after ankle implant removal. Moreover, 37.5% (3/8) patients with painless hardware developed pain after implant removal. In our series, the outcomes of implant removal was satisfactory with 94.6% of patients experiencing at least partial resolution of symptoms, and pain relief in 91.3%. The higher rate of symptom resolution in our series is likely attributed to the fact that implant removal is not routinely performed in our country, thus most of the patients who opted for removal had symptoms related to the implants.

One unique complaint in the Asian population is cold weather pain, especially those living in tropical regions where there are less seasonal variations and year-long warm temperatures. The exact mechanism for cold weather pain is unknown. It may be related to decreased local circulation and increased nerve sensitivity. We notice that this symptom presents not only in post-fixation patients, but also in patients with degenerative joint disease. This negates the correlation between the presence of metal implants and the symptom. We routinely warn our patients that cold weather pain might not be relieved with implant removal. However, in our series, cold weather pain resolved after implant removal in all 8 patients, suggesting that the presence of metallic implants may contribute to the development of this symptom.

The complication rate of implant removal has been reported as high as 40%–42%^13,14^ including wound infection, poor scar, nerve damage, and refracture. The complication rate was higher with junior surgeons^13^. Thus, implant removal was recommended to be performed by senior surgeons. It is commonly concerned that the holes left after implant removal are potential stress risers predisposing to refracture. Davison *et al*^15^ reported 27% (4/15) refractures within ten weeks of distal femur plates removal during normal functional activities. Forearm refracture rates after implant removal were reported at around 20%^16,17^. Contrary to the previous studies, our study found implant removal to be a rather innocuous procedure without any serious complications.

There were two cases with broken screws which were left retained. In both cases, variable angle locking plate was used to fix a olecranon fracture and the size of all the broken screws was 2.7mm. We believe that the smaller gauge polyaxial screws in the modern designed locked plates may predispose to a higher risk of screw breakage and retention. The strength of this study is that it is the first prospective study of routine implant removal in Singapore. Additionally, we reported cold weather pain as a unique feature of the Asian population. However, there are limitations. Firstly, this study involved a small number of patients with heterogeneous fractures. This is because implant removal is not routinely advocated and is a rare procedure locally. Secondly, patient function was not assessed as part of the study.

## Conclusion

Our study has shown that the most common reasons for elective implant removal after uneventful healing of long bone fractures were pain, prominence, and cold weather pain or discomfort. Implant removal is a safe procedure that confers a predictable relief of symptoms and satisfactory outcomes in most. Cold weather pain, although presenting in only a minority of our patients, resolved after removal of the implants.
